# CARE TRANSITION EXPERIENCES OF HISPANIC PATIENTS WITH DEMENTIA FOLLOWING EMERGENCY DEPARTMENT DISCHARGE

**DOI:** 10.1093/geroni/igae098.3820

**Published:** 2024-12-31

**Authors:** Jacqueline Sandoval, Juliette Brook, Zoe Lien, Joan Monin, Cameron Gettel, Ashley Hagaman, Ula Hwang

**Affiliations:** Yale University, New Haven, Connecticut, United States; University of Pennsylvania, Perelman School of Medicine, Philadelphia, Pennsylvania, United States; Yale University, New Haven, Connecticut, United States; Yale School of Public Health, New Haven, Connecticut, United States; Yale University School of Medicine, New Haven, Connecticut, United States; Yale University, New Haven, Connecticut, United States; New York University, New York, New York, United States

## Abstract

Older patients report fragmented emergency department (ED) care, including insufficient communication with clinicians and inadequate referrals to outpatient care and social services. These challenges are likely even greater for those with cognitive impairment (CI) and their care partners (CPs) and especially for non-English speaking populations, where language differences bring additional challenges. This study seeks to fill a gap in the literature by exploring ED discharge care transition experiences for Hispanic patients with dementia. Ten dyads - consisting of a patient with CI or dementia diagnosis and their CP - were enrolled in the ED and completed semi-structured interviews (via video or telephone) 1 week later addressing their ED and post-discharge experiences. Interviews investigated participants’ experiences in the ED and subsequent care transitions back to the community. Interviews were recorded, transcribed and coded by three research team members for thematic content, adopting a combined deductive-inductive approach until thematic saturation was reach across all dyads. Prespecified codes were adapted using the National Quality Forum’s Quality Measurement Framework on ED Transitions of Care and included eight outcomes for ED transitions of care: accessibility of services, shared decision-making, connection and alignment, care coordination, information sharing/communication, safety, healthcare utilization/costs, and experience of care. Key study findings emphasize patient and care partner priorities of shared decision-making with the ED treating team, how patients and care partners manage language barriers and cognitive impairment challenges and identifying factors that either support or hinder the quality and safety of ED discharge care and transitions back home.

